# Prognostic impact of peritumoral lymphocyte infiltration in soft tissue sarcomas

**DOI:** 10.1186/1472-6890-12-5

**Published:** 2012-02-29

**Authors:** Sveinung W Sorbye, Thomas K Kilvaer, Andrej Valkov, Tom Donnem, Eivind Smeland, Khalid Al-Shibli, Roy M Bremnes, Lill-Tove Busund

**Affiliations:** 1 Dept of Clinical Pathology, University Hospital of North Norway, 9038 Tromso, Norway; 2 Institute of Medical Biology, University of Tromso, Tromso, Norway; 3 Dept of Oncology, University Hospital of North Norway, Tromso, Norway; 4 Institute of Clinical Medicine, University of Tromso, Tromso, Norway; 5 Dept of Pathology, Nordland Central Hospital, Bodo, Norway

## Abstract

**Background:**

The purpose of this study was to clarify the prognostic significance of peritumoral lymphocyte infiltration in the capsule of soft tissue sarcomas (STS). Multiple observations in preclinical and clinical studies have shown that the immune system has a role in controlling tumor growth and progression. Prognostic markers in potentially curable STS should guide therapy after surgical resection. The immune status at the time of resection may be important, but the prognostic significance of peritumoral lymphocytes is unknown.

**Methods:**

Tissue microarrays from 80 patients with STS were constructed from duplicate cores of tissue from the tumor and the peritumoral capsule. Immunohistochemistry was used to evaluate the CD3+, CD4+, CD8+ and CD20+ lymphocytes in the tumor and the peritumoral capsule.

**Results:**

In univariate analyses, increasing numbers of CD20+ (*P *= 0.032) peritumoral lymphocytes were associated with a reduced disease free survival (DSS). In multivariate analyses, a high number of CD20+ peritumoral lymphocytes (*P *= 0.030) in the capsule was an independent negative prognostic factor for DSS. There were no such associations of lymphocyte infiltration in the tumor.

**Conclusions:**

A high density of CD20+ peritumoral lymphocytes is an independent negative prognostic indicator for patients with STS. Further research is needed to determine whether CD20 cells in the peritumoral capsule of STS may promote tumor invasion in the surrounding tissue and increase the metastatic potential.

## Background

Soft tissue sarcomas (STS) are relatively rare heterogeneous malignancies of mesenchymal origin with a high mortality rate. They comprise less than 1% of adult malignancies [1]. Approximately 50% of STS patients will succumb to their disease because of metastasis or local relapse [2]. There are several prognostic factors that determine tumour progression and, ultimately, a patients' fate. These include tumour grade, size, location, depth, histological entity, positive resection margins, and presence of local recurrence [3-9].

There are three groups of tumor infiltrating lymphocytes: (a) lymphocytes within cancer cell nests (intratumoral lymphocytes); (b) lymphocytes in the central cancer stroma (stromal lymphocytes), and; (c) lymphocytes present along the invasive margins (peritumoral lymphocytes) [10]. Soft tissue sarcomas are by definition stromal tumores. But from a biological point of view any tissue must have both parenchyma and supporting stroma. For STS it can be both internal tissue and surrounding tissue.

In a previous paper we reported the prognostic significance of lymphocyte infiltration in tumors [11]. The purpose of this study was to clarify the prognostic significance of lymphocyte infiltration in the peritumoral capsule of STS patients. This was achieved by analyzing the expression of CD3+, CD4+, CD8+ and CD20+ lymphocytes in the peritumoral capsule from 80 patients with non-gastrointestinal stromal tumors (non-GIST) STS, in relation to other clinico-pathological variables.

Stromal cells include (myo)fibroblasts, vascular cells, infiltrating leukocytes and specialized mesenchymal support cells unique to each organ microenvironment. Multiple observations in preclinical and clinical studies support a role for the immune system in controlling tumor growth and progression. However, the effects of the immune system may be contradictory because activation of the adaptive immune system may suppress malignant cells, whereas activation of various types of innate immune cells may promote tumor growth [12]. The adaptive immunity is brought about by antigen-specific T and B-lymphocytes; tumor growth is inhibited through direct killing by cytotoxic T-lymphocytes, as well as a combination of cytokine-mediated and antibody-mediated tumor cell lysis [12].

CD3 is a part of the T cell receptor (TCR) complex on mature T lymphocytes. The two major T lymphocyte subsets are (1) T helper cells (Th, CD4+) and (2) cytotoxic T cells (CTL, CD8+). CD4 is a glycoprotein expressed on the surface of T helper cells and regulatory T. CD8 is a transmembrane glycoprotein that serves as a co-receptor for the T cell receptor (TCR). Like the TCR, CD8 binds to a major histocompatibility complex (MHC) molecule, but it is specific for the class I MHC protein. CD20 is a non-glycosylated phosphoprotein expressed on the surface of all mature B-cells. CD20 is expressed at all stages of B cell development from pre-pre B cells through to memory cells (http://www.genecards.org).

It has recently become clear that analysis of the tumour stroma is paramount. This is because many anti-tumor effects operate mainly via the tumor stroma [13], and cancer infiltration by tumor reactive T-lymphocytes is required for efficient tumor eradication [14]. However, cancer cells can evade the immune system in many ways including suppression of cytotoxic T-cells by regulatory T-cells and by accumulation of myeloid suppressor cells [14-17].

Many studies have been designed to investigate the prognostic factors of STS using immunohistochemical methods [18]. Most of the published data has focused on the expression of markers for cell kinetics and regulatory proteins of the cell cycle. There are few studies of the prognostic impact of lymphocytes in STS [11].

## Methods

The National Cancer Data Inspection Board and The Regional Committee for Research Ethics approved the study. The material was collected from our approved biobank for paraffin-embedded materials and slides. The Regional Committee confirmed that written consent from the patients for their information to be stored in the hospital database and used for research was not needed. This is because most of the material was more than 10 years old, and most of the patients were dead. The data was analyzed anonymously.

Primary tumor tissue from patients diagnosed with STS at the University Hospital of North Norway (UNN) from 1973 to 2006 and the Hospitals of Arkhangelsk region, Russia, from 1996-2006 was used in this retrospective study. 496 potentially suitable patient records were identified from the hospital database but only 249 of these were eligible because in they had complete medical records and adequate paraffin-embedded tissue blocks. In 80 of these cases it was possible to obtain tissue from the peritumoral capsule for TMA.

This report includes follow-up data for 59 Norwegian and 21 Russian patients. The median follow-up was 38 (range 0-392) months. Complete demographic and clinical data were collected retrospectively. Formalin-fixed and paraffin-embedded tumor specimens were obtained from the archives of the Departments of Pathology at UNN and Arkhangelsk. The tumors were graded according to the French Fèdèration Nationales des Centres de Lutte Contre le Cancer (FNCLCC), [WHO Tumors of Soft Tissue and bone, 2002]. Wide resection margins were defined as wide local resection with free microscopic margins or amputation of the affected limb or organ. Non-wide resection margins were defined as either marginal or intra-lesional resection margins, or no surgery.

The histology of all soft tissue sarcoma cases was reviewed according to modern classification (WHO, 2002) by two pathologists (AV and SWS). For the Russian material there were made new slides of all the paraffin blocks. For the Norwegian material new slides were made when necessary. All the biopsies were immunostained with CK, CD117, Actin, SMA, VIM and CD34. Some slides were also stained with S100 if necessary to rule out differential diagnosis. Other molecular methods where not considered as necessary for differential diagnostics, but in some cases PCR or FISH were performed in the initial diagnostics. Ten of the initial diagnoses were revised due to changes of classification system and appearance of new entities as GIST. All carsinosarcomas, endometrial sarcomas, carcinomas and lymphomas were excluded.

There is some lymphocyte heterogeneity in tumor and capsule which gives variation on lymphocyte counts within tumours. TMAs are therefore not useful to estimate the prognosis of every single patient, but in large groups the mean expression will be representative for the group. In average the heterogeneity will not be a problem if the sample size is large enough. Usually 60 samples are considered to be enough to get a representative material. We had two cores from tumor and two cores from peritumoral capsule from 80 patients. If we had used three cores from the tumor and three cores from the peritumoral capsule we would secure the representativeness even more and reduce sampling errors, but we did not want to use too much tissue from the donor blocs.

### Microarray construction

Tissue microarrays (TMAs) were constructed for high-throughput molecular pathology research [19-21]. The slides were evaluated by two pathologists using a light microscope to identify the peritumoral capsule (AV and SWS). The most representative areas of the tumor and the peritumoral capsule were carefully selected and marked on the hematoxylin and eosin (HE) slides for the corresponding donor blocks and sampled for the tissue microarray collector blocks. Soft tissue sarcomas are by definition stromal tumores. But from a biological point of view any tissue must have both parenchyma and supporting stroma. For STS it can be both internal tissue and surrounding tissue. Therefore we have chosen to focus on the tumor capsule (pseudocapsule). We defined the peritumoral capsule as the 1-3 mm thick layer of connective tissue surrounding the parenchyma of the tumor. In high grade tumors there was no typical capsule, but in several of the cases it was a desmoplastic reaction in the surrounding tissues which was also obtained as capsule. The TMAs were assembled using a tissue-arraying instrument (Beecher Instruments).

Studies suggest that punching multiple 0.6 mm cores from different regions captures the heterogeneity of the capsule more accurately than single 2 to 4 mm cores [21]. Hence, we used two 0.6-mm cores of the tumor and two cores from the peritumoral capsule that were taken from different areas and selected to be as representative as possible. To include all core samples, sixteen tissue array blocks were constructed. Multiple 4-μm sections were cut with a Micron microtome (HM355S) and specific antibodies were stained for immunohistochemistry (IHC).

### Immunohistochemistry (IHC)

The applied antibodies were subjected to in-house validation by the manufacturer for IHC analysis on paraffin-embedded material. Ventana Benchmark, XT automated slide stainer (Ventana Medical System, France) was used for IHC. Sections were de-paraffinized with xylene and rehydrated with ethanol. Antigen retrieval was performed by placing the specimens in 0.01 M citrate buffer at pH 6.0 and twice exposing to microwave heating for 10 minutes at 450 W. The DAKO Envision + System-HRP (DAB) kit was used as endogen peroxidase blocking. As negative staining controls, the primary antibodies were replaced with the primary antibody diluents. Primary mouse monoclonal antibodies were incubated for 16 minutes (CD20, clone L26 Ventana); 20 minutes (CD4, clone 1 F6 Novocastra, dilution 1:5); and 32 minutes (CD8, clone 1A5 Ventana) at room temperature. The Ventana antibodies were pre-diluted by the manufacturer. Biotinylated goat anti-mouse IgG and mouse anti-rabbit IgM were used as secondary antibodies. The DAB was used to make the antigens visible. This was followed by application of liquid diaminobenzidine and substratechromogen, yielding a brown reaction product at the site of the target antigen. Finally, slides were counterstained with hematoxylin to make the nuclei visible. TMA staining was performed in a single experiment for each antibody, including negative controls.

### Scoring of IHC

**Figure 1:**
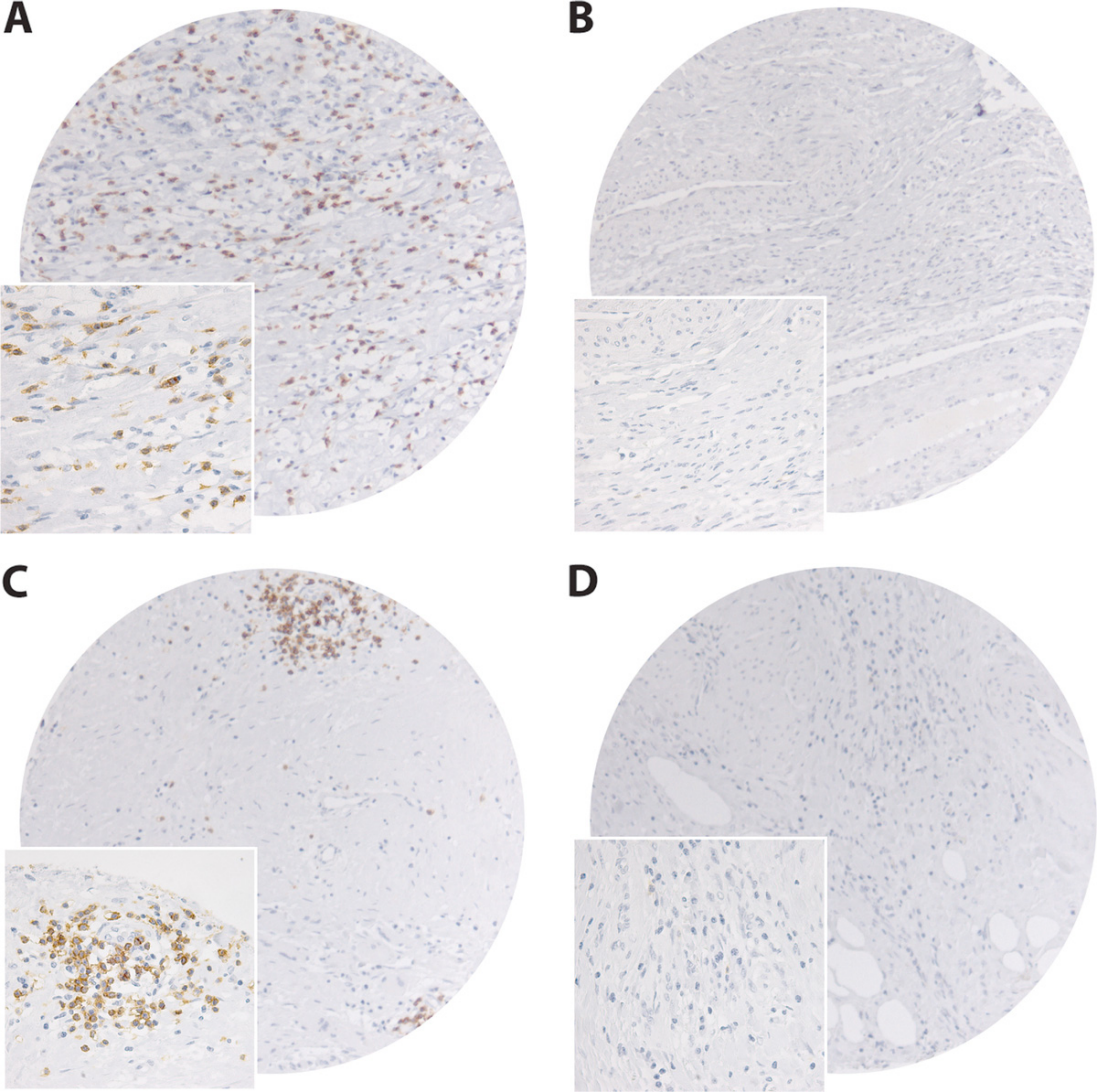
**Pictures of tissue cores**. IHC microscopic pictures of TMA of soft tissue sarcomas representing different scores for CD8+ and CD20+ lymphocytes in peritumoral capsule. **(A) **CD8 high score; **(B) **CD8 low score; **(C) **CD20 high score; **(D) **CD20 low score. Original magnification, ×100 and ×400.

The ARIOL imaging system (Genetix, San Jose, CA) was used to scan the slides for antibody staining of the TMAs. The specimens were scanned at a low resolution (1.25 ×) and high resolution (20 ×) using an Olympus BX 61 microscope with an automated platform (Prior). The slides were loaded in the automated slide loader (Applied Imaging SL 50). Representative and viable tissue sections from the peritumoral capsule were scored semi-quantitatively for lymphocyte membrane staining. For each core the tissues were scored manually on a computer screen as: 0 (no cells), 1 (1-5 cells), 2 (6-19 cells), or 3 (20+ cells) (Figure 1). We have included all lymphocytes located within both inner and outer parts of the capsule. The score for each patient was based on the mean scorings of cores from the one or several biopsies. To achieve maximum reproducibility in all cases every staining was dichotomised (low and high expression). High expression was defined as mean score ≥ 1 for all the immunomarkers (CD3, CD4, CD8 and CD20).

Of the 80 patients, we had three missing for immunostaining with CD20. Of the 77 patients with valid score, a total of 57 patients had the same score in both cores, and 20 had different scores. When the two cores from each tumor had different expression, we used the mean score. If the scores were 0 and 1, the mean score was 0.5. If the scores were 1 and 2, the mean score was 1.5. In the first case, the mean score was < 1 (low expression). In the second case, the mean score was > 1 (high expression).

All samples were made anonymous and independently scored by two pathologists (AV and SWS). Where there was disagreement, the slides were re-examined and a consensus was reached by the observers. When assessing a variable for a given score, the scores of the other variables, and the outcome, were hidden from the observers.

### Statistical methods

Statistical package SPSS (Chicago, IL), version 16 was used for all statistical analyses. The immunohistochemistry scores from each observer were compared for inter-observer reliability using a two-way random effect model with absolute agreement definition. The intra-class correlation coefficient (reliability coefficient) was obtained from these results.

The Chi-square test and Fishers Exact test were used to examine the association between molecular marker expression and various clinicopathological parameters. Univariate analyses were carried out using the Kaplan-Meier method, and statistical significance between survival curves was assessed using the log rank test. Disease-specific survival (DSS) was determined from the date of histological-confirmed STS diagnosis.

Multivariate analysis was carried out using the Cox proportional hazards model to assess the independent impact on survival of each pre-treatment variable in the presence of other variables. Only variables of significant value from the univariate analysis were entered into the Cox regression analysis. Probability for stepwise entry and removal was set at 0.05 and 0.10, respectively. The significance level used was *p *< 0.05.

## Results

### Clinicopathological variables

Demographic, clinical, and histopathological variables are shown in Table 1. Patient age range was 0-91 years (mean 56 years, median 59.5 years), and 44% of the patients were males. The non-GIST soft tissue sarcomas (STS) comprised: 20 undifferentiated pleomorphic sarcoma, 21 leiomyosarcoma, 7 angiosarcoma, 6 liposarcoma, 6 rhabdomyosarcoma, 6 synovial sarcoma, 5 malignant peripheral nerve sheath tumors (MPNST), 4 malignant fibroblastic/myofibroblastic tumors, and 5 other STS. There were 15 low grade (19%) and 65 high grade (81%, FNCLCC grade 2 and 3) STS.

**Table 1:** Clinicopathologic variables and surival.

Characteristic	Patients (n)	Patients (%)	Median survival (months)	5-Year survival (%)	P
**Age**					
≤ 20 years	7	9	NR	57	0.479
21-60 years	36	45	63	53	
> 60 years	37	46	36	44	
**Sex**					
Male	35	44	63	50	0.847
Female	45	56	57	49	
**Nationality**					
Norwegian	59	74	38	48	0.646
Russian	21	26	127	52	
**Histology**					
Undifferentiated pleomorphic sarcoma	20	25	127	57	0.025
Leiomyosarcoma	21	26	67	52	
Angiosarcoma	7	9	15	29	
Liposarcoma	6	8	36	50	
Rhabdomyosarcoma	6	8	32	50	
Synovial sarcoma	6	8	31	50	
MPNST	5	6	NR	80	
MF/MFT	4	5	15	50	
Other STS	5	6	11	0	
**Tumor localization**					
Extremities	31	39	123	61	0.447
Trunk	15	19	32	47	
Retroperitoneum	11	14	57	42	
Head/Neck	5	6	15	20	
Pelvis	18	23	22	41	
**Tumor size**					
≤ 5 cm	26	33	67	54	0.800
5-10 cm	33	41	31	45	
> 10 cm	20	25	80	53	
Missing	1	1			
**Malignancy grade FNCLCC**					
1	15	19	NR	70	0.007
2	31	39	80	61	
3	34	43	15	29	
**Tumor depth**					
Superficial	4	5	NR	100	0.046
Deep	76	95	32	46	
**Operated**					
Yes	73	91	75	54	< 0.001
No	7	9	5	0	
**Surgical margins**					
Wide	39	49	NR	67	< 0.001
Non-wide	41	51	18	32	
**Metastasis at diagnosis**					
No	65	81	80	57	< 0.001
Yes	15	19	11	14	
**Chemotherapy**					
No	59	74	36	46	0.147
Yes	21	26	NR	57	
**Radiotherapy**					
No	60	75	38	47	0.775
Yes	20	25	63	55	

Prognostic clinicopathologic variables as predictors for disease-specific survival in soft tissue sarcomas with peritumoral capsule (univariate analysis, log rank test), N = 80 Abbreviations: MF/MFT, malignant fibroblastic/myofibroblastic tumors; MPNST, malignant peripheral nerve sheath tumor; STS, soft tissue sarcomas; NR, not reached Ninety-one percent (n = 73) underwent surgery. The 5-year survival with non-wide resection margins was 32% and with wide resection margins it was 67%.

### Interobserver variability

There was good reproducibility between the two investigating pathologists. Their scoring agreement was tested for CD8 and CD20. The intra-class correlation coefficients (reliability coefficients, r) obtained from these results were 0.90 for CD8 (*P *< 0.001) and 0.90 for CD20 (*P *< 0.001).

### Univariate analysis

Histological entity, malignancy grade, tumor depth, surgery, surgical margins and metastasis at time of diagnosis were all significant indicators for disease-free survival (DSS) in univariate analyses (Table 1).

**Figure 2:**
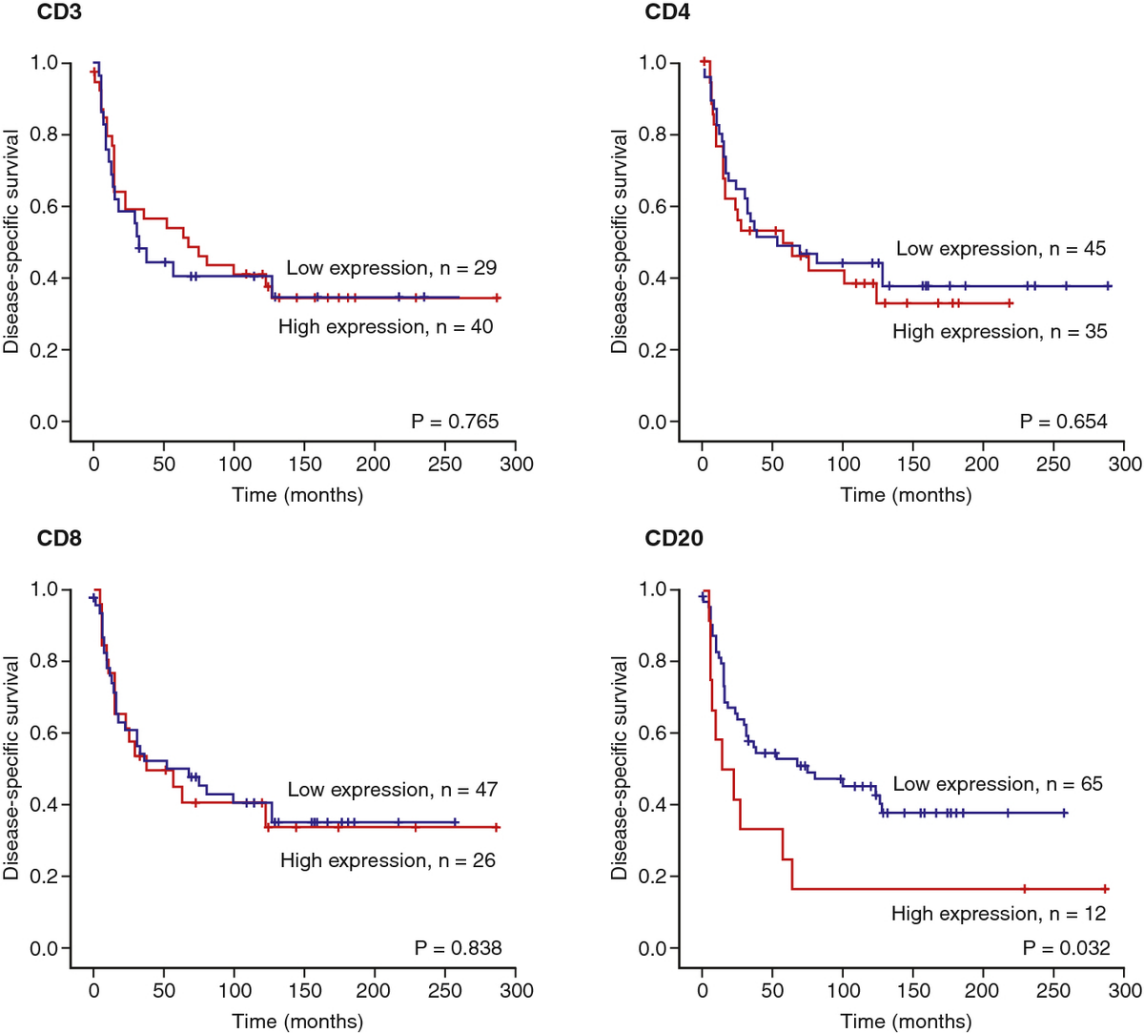
**Survival curves**. Disease-specific survival curves for CD3+, CD4+, CD8+ and CD20+ lymphocytes in peritumoral capsule in soft tissue sarcomas.

Increasing numbers of CD20+ (*P *= 0.032) lymphocytes in the peritumoral capsule were significantly associated with a reduced DSS. No such relationship was apparent for CD3+, CD4+ and CD8+ lymphocytes (Table 2 Figure 2). In subgroup analysis increasing numbers of CD20+ lymphocytes were significantly associated with a reduced DSS in patients over 60 years (*P *< 0.001), patients with high grade tumors (*P *= 0.012), and patients without wide surgical resection margins (*P *= 0.003) (data not shown). There were no such associations of lymphocyte infiltration in the tumor (Table 2).

**Table 2:** Expression of markers and survival.

			Tumor				Peritumoral capsule			
**Marker expression**	**Patients (n)**	**Patients (%)**	**Median survival (months)**	**5-Year survival (%)**	**P**	**Patients (n)**	**Patients (%)**	**Median survival (months)**	**5-Year survival (%)**	**P**

**CD 3**										
Low	56	70	80	54	0.207	29	36	32	47	0.756
High	19	24	27	36		40	50	67	56	
Missing	5	6				11	14			
**CD 4**										
Low	64	80	57	49	0.887	45	56	52	51	0.654
High	14	18	27	50		35	44	57	53	
Missing	2	3				10	12			
**CD 8**										
Low	64	80	80	54	0.198	47	59	67	52	0.838
High	14	18	15	36		26	32	38	50	
Missing	2	3				7	9			
**CD 20**										
Low	56	70	63	50	0.880	65	81	75	55	0.032
High	22	28	32	50		12	15	13	33	
Missing	2	3				3	4			

Peritumoral capsule lymphocyte subsets and their prediction for disease-specific survival in patients with soft tissue sarcomas (univariate analysis; log-rank test), N = 80

### Multivariate analyses

Significant demographic, clinicopathological, and lymphocyte infiltrate variables from the univariate analyses were entered into the multivariate Cox regression analysis. We did not undertake a multivariate analysis of lymphocyte counts with CD20 in the tumor, because in this dataset there was no statistical significance on prognosis of CD20 counts in the tumor. A high number of CD20 lymphocytes in the peritumoral capsule had an independent negative survival impact (HR 2.7, CI95% 1.10-6.60, *P *= 0.030). Other independent prognostic variables were malignancy grade (*P *= 0.001), surgical operation (*P *< 0.010), and surgical margins (*P *< 0.001), (Table 3).

**Table 3:** Results of Cox regression analysis in peritumoral capsule summarizing prognostic factors, N = 80

Factor	Hazard Ratio	95% CI	P
**Tumor size**			0.780*
≤ 5 cm	1.000		
5-10 cm	1.328	0.601-2.933	0.484
> 10 cm	1.268	0.505-3.186	0.613
**Malignancy grade FNCLCC**			0.001
1	1.000		
2	3.481	0.899-13.470	0.071
3	8.448	2.285-31.230	0.001
**Operated**			
Yes	1.000		
No	5.594	1.504-20.812	0.010
**Surgical margins**			
Wide	1.000		
Non-wide	3.633	1.768-7.463	< 0.001
**Metastasis at time of diagnosis**			
No	1.000		
Yes	2.088	0.977-4.463	0.057
**CD20 + cells**			
Low	1.000		
High	2.696	1.102-6.598	0.030

* Overall significance as a prognostic factor

## Discussion

In this study, we evaluate the prognostic impact of CD3+, CD4+, CD8+ and CD20+ lymphocytes in the peritumoral capsule and associations with clinico-pathological variables in 80 non-GIST STS. To our knowledge, this is the first study of the association between lymphocyte infiltration in the peritumoral capsule and DSS in STS patients. Interestingly, high numbers of CD20+ cells in the capsule was an independent negative prognostic factor for DSS.

Tumor infiltrating CD20+ B-cells are correlated with an improved DSS in lung cancer, prostate cancer, ovarian cancer and STS [11,22-25]. Tumor infiltrating lymphocytes are considered to be an indication of the host immune reaction to tumor antigens [26]. Pelletier studied several prognostic factors in 113 NSCLC tumors, including peritumoral B (CD20+) and T (CD43+) lymphocytes, and found B cells to be associated with an improved DSS [25]. Peritumoral lymphocytes in epithelial tumors may have different significance from lymphocytes in the capsule in stromal tumors. In our study, high numbers of CD20 cells in the capsule were an independent negative prognostic factor for DSS in STS. Tumor-associated inflammation is a chronic process which is often unfavorable for the host [27]. The precipitation of immunoglobulin in the tissue may have a stimulatory effect on the innate immune cells, which may enhance tumor growth by secreting mediators which stimulate angiogenesis [12]. Many immune cells found in the tumor microenvironment may be associated with the tissue disruption caused by inflammatory agents or they may be a response to tumor growth [27]. In fact, de Visser et al. showed in an animal model that B lymphocytes are required for establishing chronic inflammatory states that promote de novo carcinogenesis [28]. However, it is possible that B regs might accumulate at the tumor site.

There has been significant progress in cancer immunotherapy during the last decade [27]. Progress in understanding the molecular mechanisms that govern immune activation, as well as the mechanisms used by tumor cells to evade surveillance by the immune system, are advancing the development of immune-mediated therapies that could be used against a range of human cancers [29].

Evidence on the role of the immune system in limiting tumor growth and progression is linked to observations indicating a positive correlation between the presence of tumor infiltrating CD8+ T-cells and good prognosis in various types of cancer [30]. CD8+ cells in malignant tumors have been associated with an improved DSS in: non-small cell lung carcinoma; carcinomas of the endometrium, bile duct, colon, oesophagus, and urothelium; uveal melanoma; and follicular lymphoma [22,31-40]. Tumor infiltrating CD8+ lymphocytes were not associated with improved survival in STS [11]. However, in mice with solid sarcoma EGF-SEA, CD4+, CD8+ and SEA-reactive T lymphocytes strongly suppressed tumor growth [41].

The role of CD8+ lymphocytes in the peritumoral capsule of STS is unknown. The location of infiltrating lymphocytes may be important. There are major differences between 1) lymphocytes within cancer cell nests in carcinomas (epithelial lymphocytes); 2) lymphocytes present along the invasive margins (peritumoral lymphocytes); and, 3) lymphocytes in the capsule of stromal tumors such as STS. Galon et al. investigated the relationship between the type, density, and location of immune cells within tumors and the clinical outcome of patients with colorectal carcinomas. They found that patients with low densities of CD3+ cells, CD8+ and CD45RO + memory T cells in both the centre of the tumor and the invasive margins had a very poor prognosis [32]. High expression of tumor infiltrating CD3+ T-cells is associated with good prognosis in various types of cancers [30]. High number of CD8+ cytotoxic T-lymphocytes infiltrating primary and metastatic ovarian cancer sites are associated with improved prognosis [42]. In our material there was no significant correlation between DSS and CD3+ or CD8+ infiltrating cells in the capsule.

The role of CD4+ T and B lymphocytes is controversial in many cancers including STS [30]. In the absence of CD8+ cytotoxic T cells CD4+ cells are critical, and sufficient for NKT cell-dependent rejection of experimental tumours [43]. There was a positive correlation between a high density of CD4+ and CD8+ lymphocytes in stroma and improved disease-specific survival in non-small cell lung cancer [22]. In our material we did not find any significant correlation between DSS and CD4+ infiltrating cells in the capsule.

In a previous paper we reported that high density of CD20+ lymphocytes in tumor of STS is an independent positive prognostic indicator in 108 patients with wide resection margins [11], but not when the 141 patients with non-wide resection margins were included in the analysis. In this paper only 39 patients with material from the peritumoral capsule had wide resection margins and there was no effect on prognosis of CD20 counts in tumor. However, a high density of CD20+ lymphocytes in the peritumoral capsule is a negative prognostic indicator for patients with STS, independent of resection margins.

## Conclusions

High density of CD20+ peritumoral lymphocytes is an independent negative prognostic indicator for patients with STS. These data need to be verified in larger-scale studies. Furthermore, experimentally designed research is required in order to define whether CD20 cells in the peritumoral capsule of STS promote tumor invasion and/or metastasis.

## Competing interests

The authors declare that they have no competing interests.

## Authors' contributions

SWS, TK, AV, TD, RMB and LTB participated in the design of the study. TK and AV collected clinical information. SWS and AV reviewed all the histological diagnosis, histological grading, selected and marked the slides for TMA construction. SWS, TK and AV performed the experiments. SWS, TK, AV, TD, RMB and LTB performed the statistical analysis. SWS, TK, AV, TD, ES, KAS and LTB contributed reagents/materials/analysis tools. SWS, TD, ES, KAS, RMB and LTB drafted the manuscript. All authors read and approved the final manuscript.

## Pre-publication history

The pre-publication history for this paper can be accessed here:

http://www.biomedcentral.com/1472-6890/12/5/prepub
